# Supplementary material for: Oligonucleotide‐induced alternative splicing of serotonin 2C receptor reduces food intake
Source: EMBO Mol Med. 2016 Jul 12;8(8):878–94. doi: 10.15252/emmm.201506030 (PMC4967942; doi:10.15252/emmm.201506030)
Supplement: Supplementary file 1 — Expanded View Figures PDF [file EMMM-8-878-s001.pdf]

Expanded View Figures

A

```
human     MVNLRNAVHSFLVHLIGLLVWQCDISVSPVAAIVTDIFNTSDGGR-FKFPDGVQNWPALS
mouse     MVNLGTAVRSLLVHLIGLLVWQFDISISPVAAIVTDTFNSSDGGRLFQFPDGVQNWPALS
          **** .**:*:*********** ***:********* **:***** *:************

human     IVIIIIMTIGGNILVIMAVSMEKKLHNATNYFLMSLAIADMLVGLLVMPLSLLAILYDYV
mouse     IVVIIIMTIGGNILVIMAVSMEKKLHNATNYFLMSLAIADMLVGLLVMPLSLLAILYDYV
          **:*********************************************************

human     WPLPRYLCPVWISLDVLFSTASIMHLCAISLDRCISSYPCDWTEGRRKGVREQHDVRAQR
mouse     WPLPRYLCPVWISLDVLFSTASIMHLCAISLDRSFSSYPCDWTEGRKQSVRE--------
          *********************************.:***********::.***

human     PKFRSYWVLRSFLHTADDYGDYVLPDHLRSAPTSFDVTARPHRGTAWTKSGFPEVLQEEY
mouse     ------------------------------------------------------------

human     GRGRELCKP
mouse     ---------
```

B

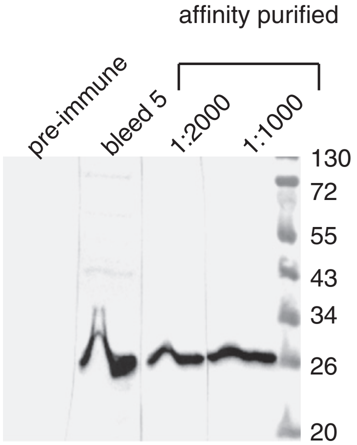

Figure EV1. Antiserum against the truncated form of the serotonin 2C receptor (RNA1).

A Sequence comparison between the human and mouse truncated 5HT2C. The amino acids encoded by exon VI are in bold. The peptide sequence used to generate the rabbit antiserum is underlined.

B Characterization of the antiserum. HEK293 cells were transfected with a cDNA encoding the human RNA1. A high titer developed after five rounds of immunization (bleed 5). The human protein has a predicted molecular weight of 28 kd. The mouse protein has a predicted molecular weight of 19 kD and runs slightly slower, possibly due to posttranslational modifications common for membrane proteins.

Source data are available online for this figure.

 © 2016 The Authors

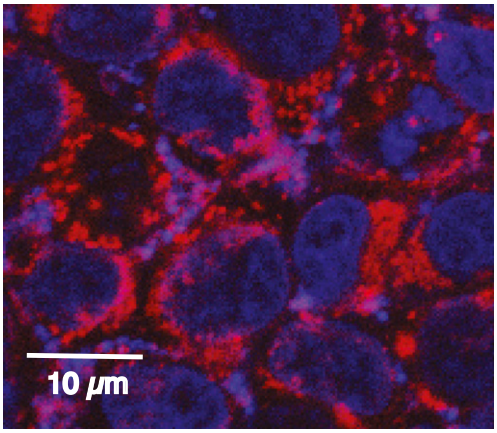

Figure EV2. Uptake of Cy3-labeled oligo#5 in HEK293 cells.
The oligo (red) was added to the cell culture medium, and 3 h later, the medium was changed and cells were visualized. The nuclei are stained with DAPI (blue).

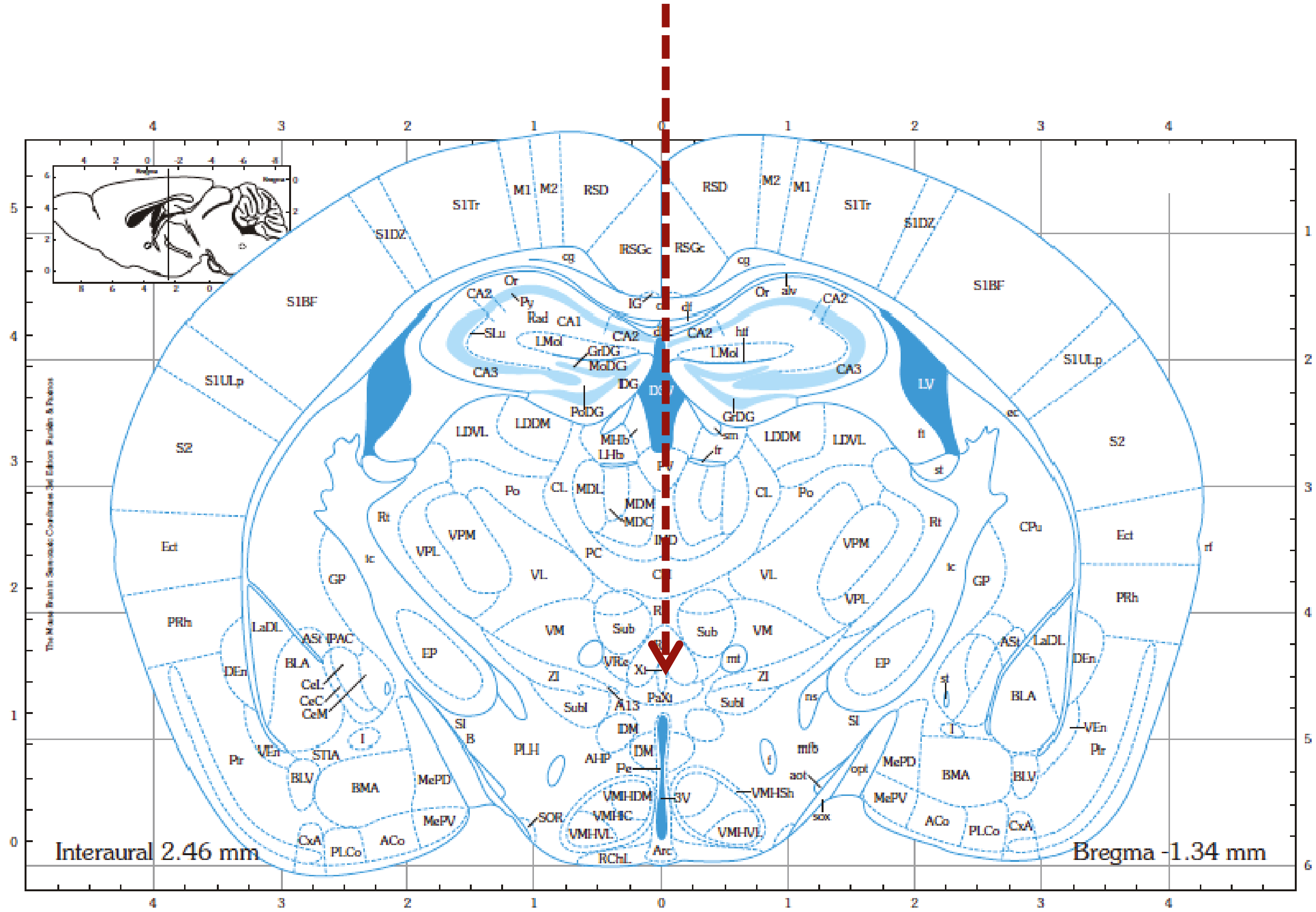

Figure EV3. Overview of the injection strategy using a coronal section of the area used.
The arrow indicates the guide cannula for injection into the third ventricle. The injection cannula is inserted into the 3rd ventricle.

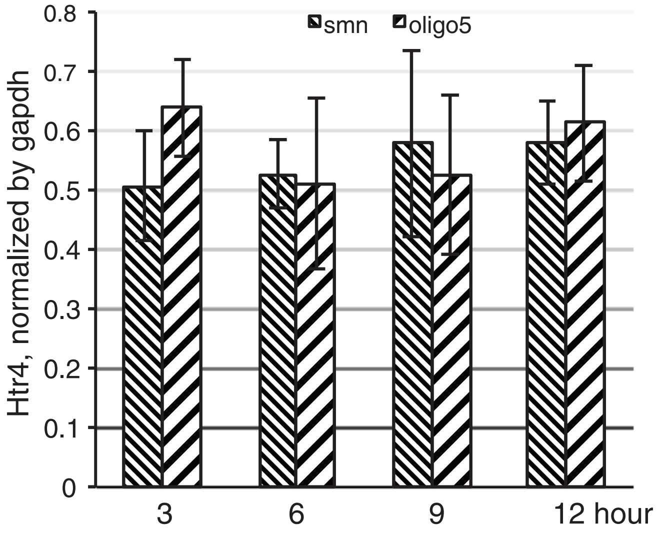

Figure EV4. Effect of oligo#5 on Htr4 expression.
The same RNA as in Fig 5E and G was used and was analyzed by qPCR using primers that detect Htr4. Injection of 2 μg of an oligo against human SMN2 was used as a unspecific control, since there is no SMN2 sequence in mice. *n* = 4, error bars indicate standard error.

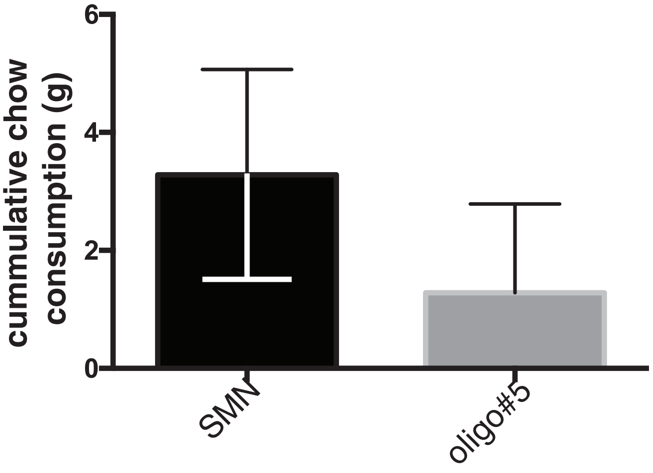

Figure EV5. Effect of oligo#5 on food uptake in MCR4 KO mice.
MCR4 knockout mice were treated as wild-type mice (Fig 5E) and were in injected with 1 μg oligo#5. There was no significant difference in food uptake after 12 h (P = 0.1965, t = 1.5.48, df = 4, n = 6 paired t-test). Error bars indicate standard deviation.

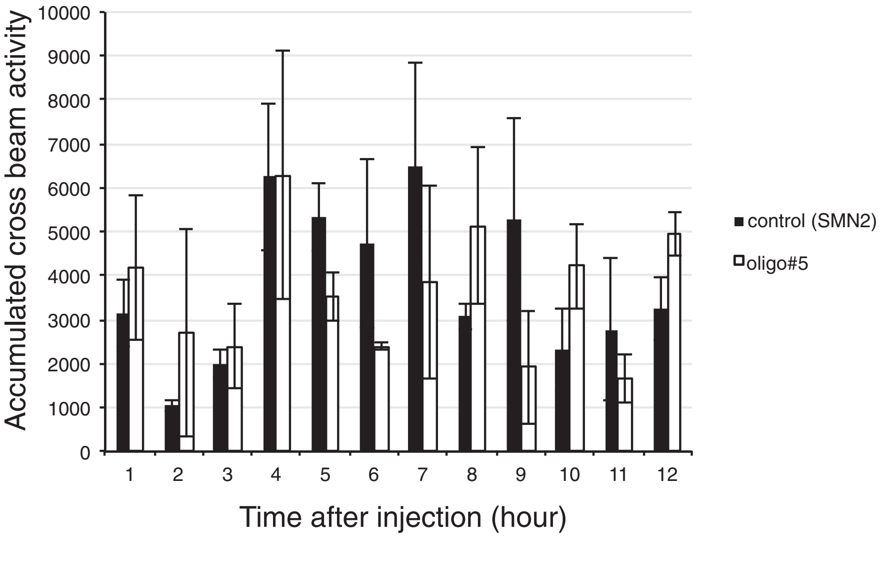

Figure EV6. Activity of mice after oligo#5 injection.
The activity of mice receiving oligo#5 or control SNM oligo was measured using the numbers of beam-crossing in metabolic cages. There is no statistical significant difference between groups. *n* = 12, error bars indicate standard deviation.

 © 2016 The Authors
