# Supplementary material for: Oligonucleotide‐induced alternative splicing of serotonin 2C receptor reduces food intake
Source: EMBO Mol Med. 2016 Jul 12;8(8):878–94. doi: 10.15252/emmm.201506030 (PMC4967942; doi:10.15252/emmm.201506030)
Supplement: Supplementary file 2 — Source Data for Expanded View [file EMMM-8-878-s006.pdf]

Figure EV1 B

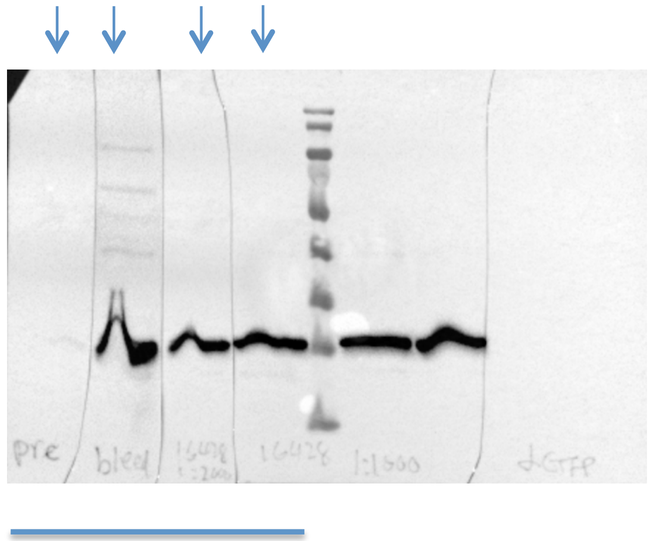


Protein loaded on one gel, membrane cut into stripes and tested with different antiserum/bleed
