# Supplementary figures and images for: Oligonucleotide‐induced alternative splicing of serotonin 2C receptor reduces food intake
Source: EMBO Mol Med. 2016 Jul 12;8(8):878–94. doi: 10.15252/emmm.201506030 (PMC4967942; doi:10.15252/emmm.201506030)

Figure 1

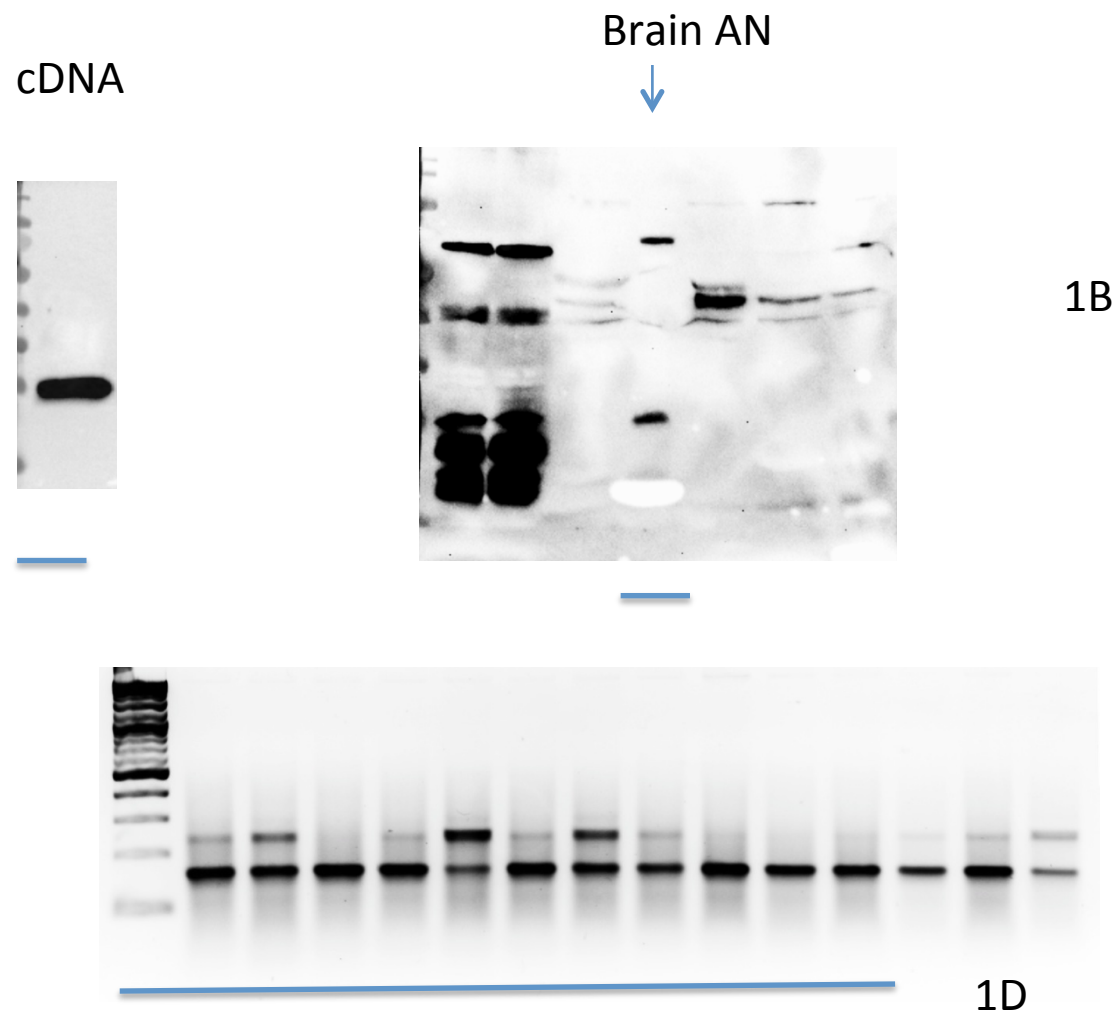

Supplement: Supplementary file 4 — Source Data for Figure 1 [file EMMM-8-878-s002.pdf]

2A

Figure 2

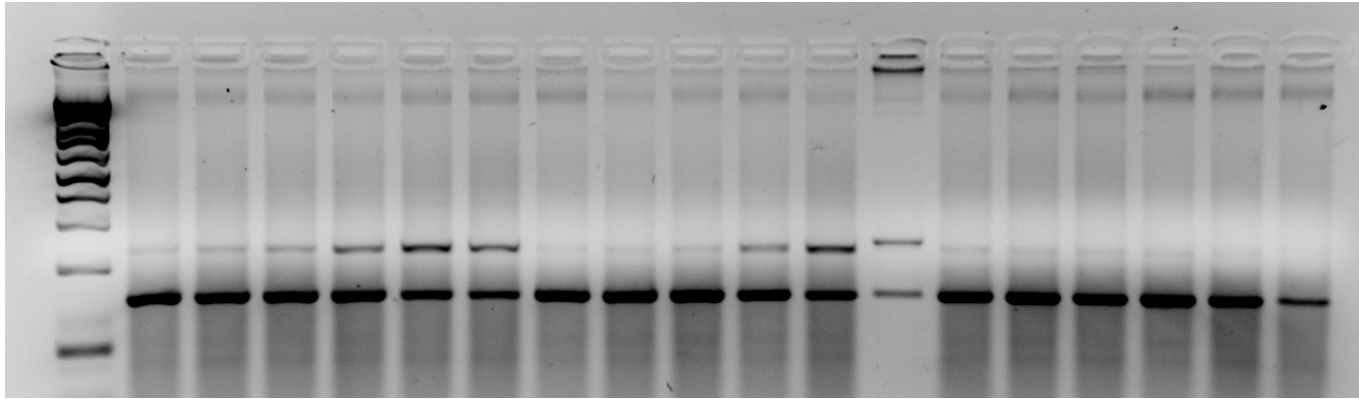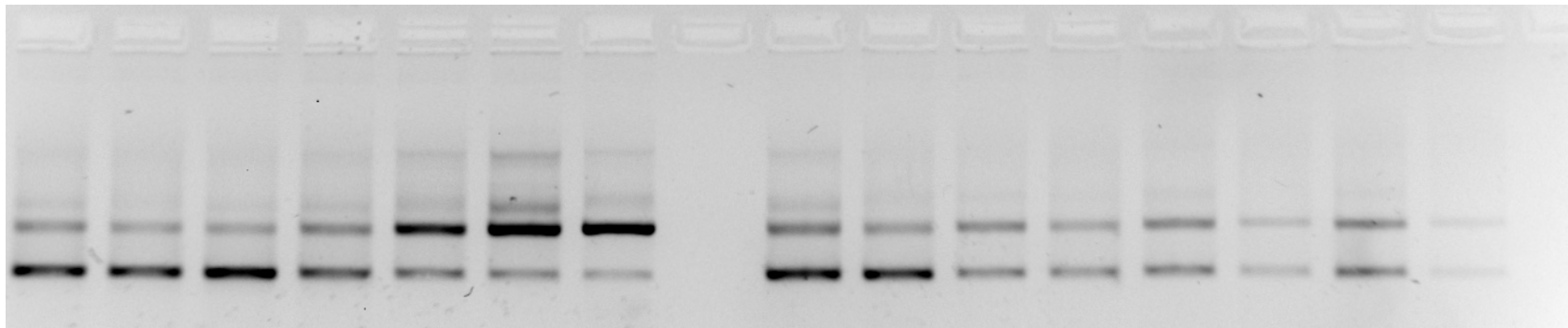

2A

Figure 2

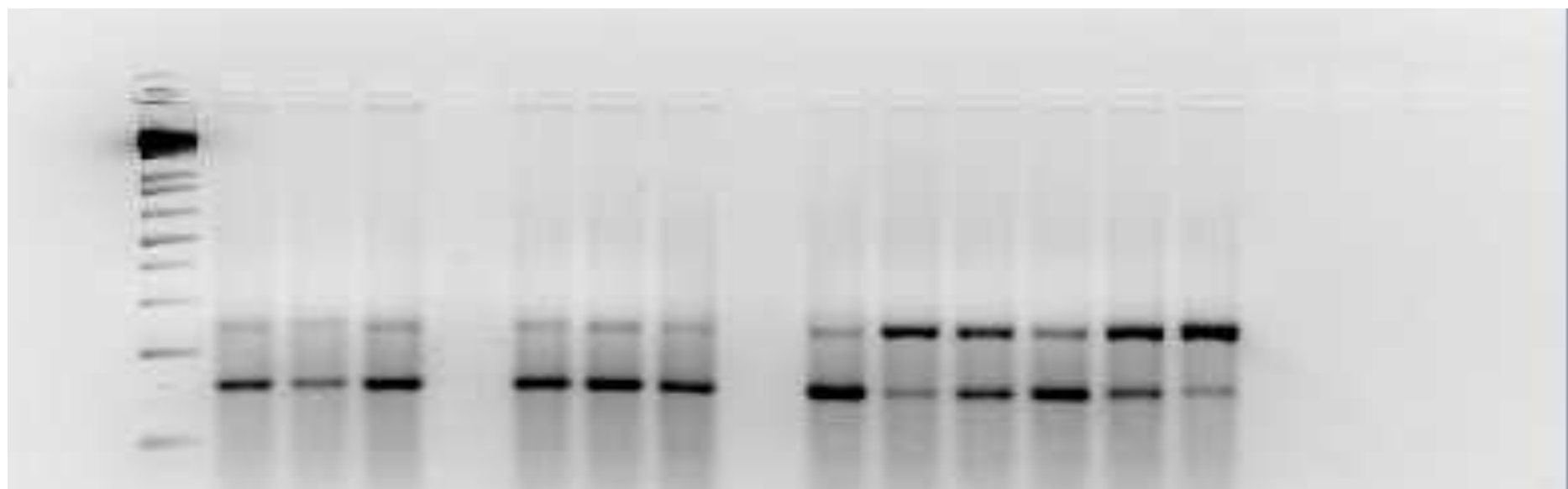

---

2C

Figure 2

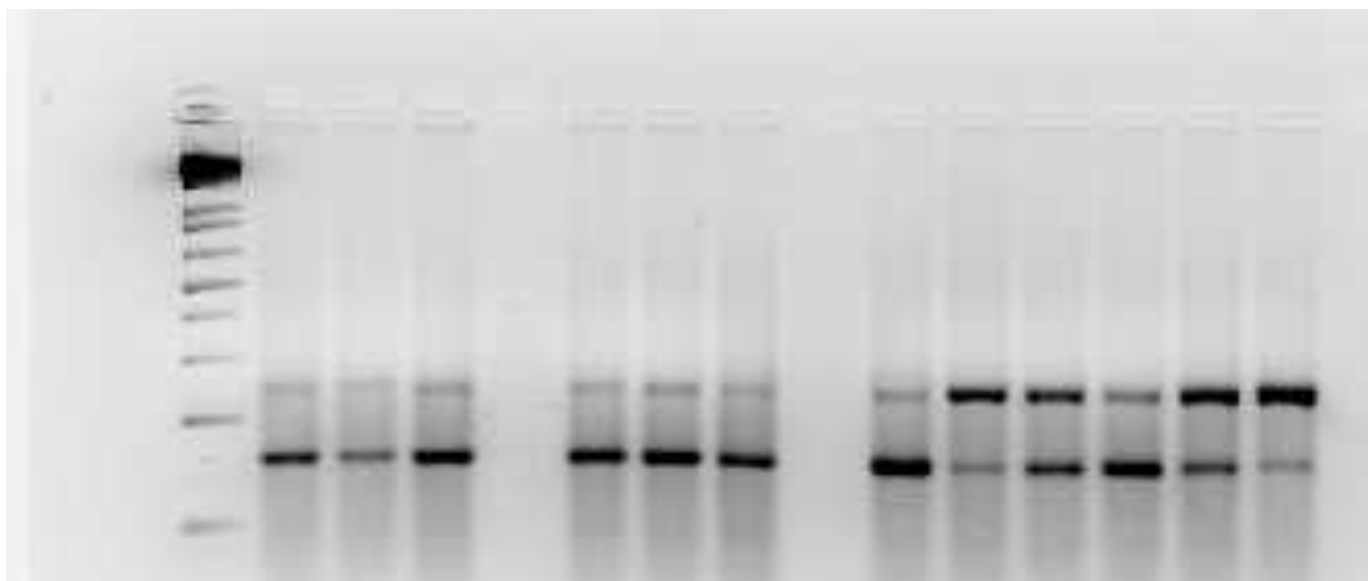

—

2E

Supplement: Supplementary file 5 — Source Data for Figure 2 [file EMMM-8-878-s003.pdf]

Figure 3

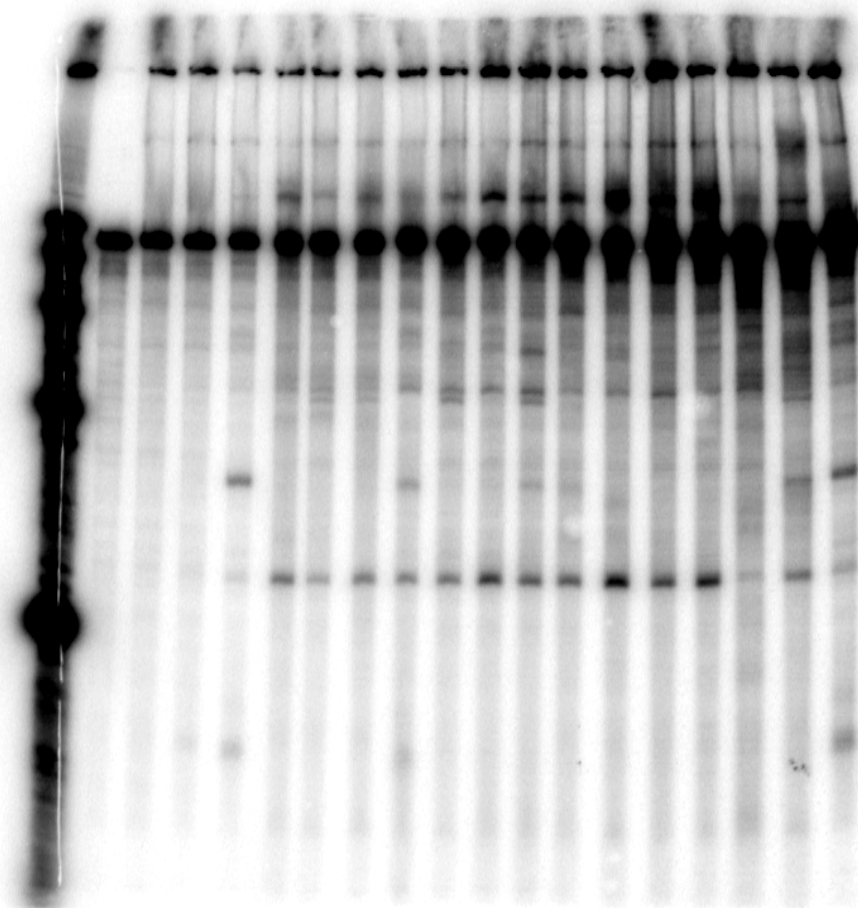

Supplement: Supplementary file 6 — Source Data for Figure 3 [file EMMM-8-878-s004.pdf]
