# Supplementary material for: Oligonucleotide‐induced alternative splicing of serotonin 2C receptor reduces food intake
Source: EMBO Mol Med. 2016 Jul 12;8(8):878–94. doi: 10.15252/emmm.201506030 (PMC4967942; doi:10.15252/emmm.201506030)
Supplement: Supplementary file 7 — Source Data for Figure 4 [file EMMM-8-878-s005.pdf]

Figure 4

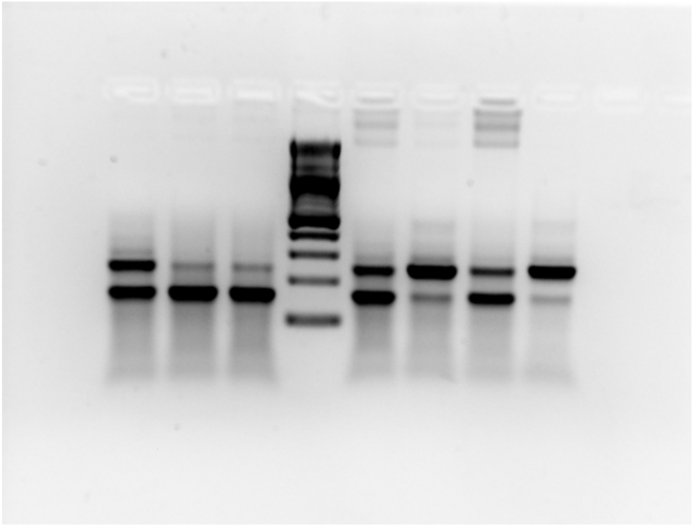

4B

Figure 4

Figure 4C, top

4C, left panel

Anti-RNA1

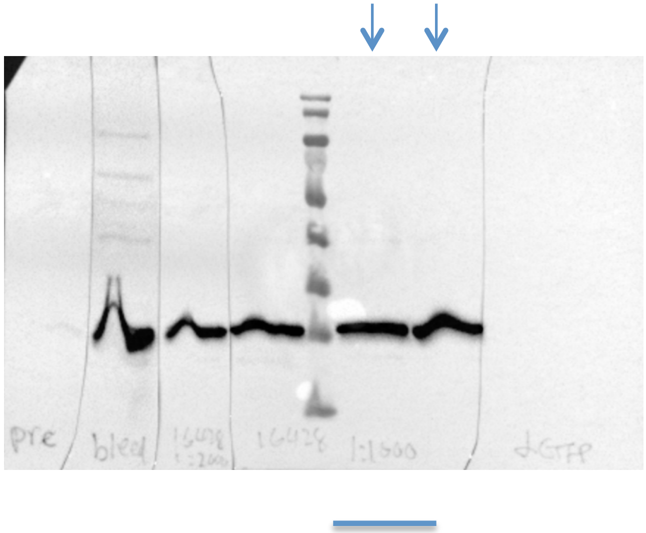

4C, right panel

Anti-RNA1

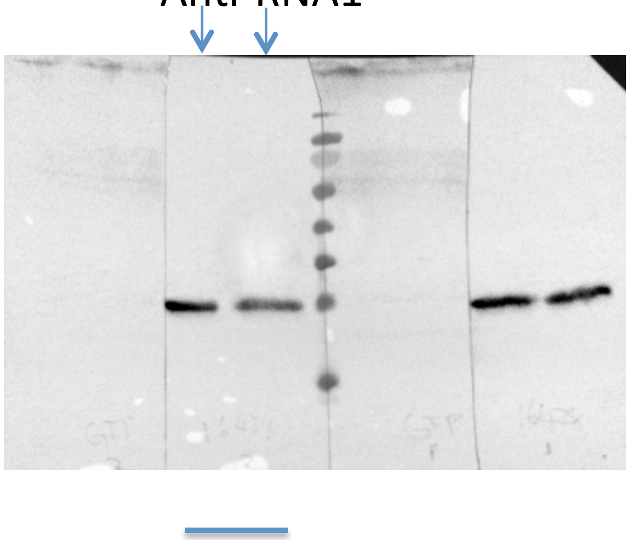

Protein was run on one gel,
membranes were cut with scissors,
and blotted with two different antisera

Figure 4

Figure 4C, bottom

4C, left

Reblot: anti-beta-actin

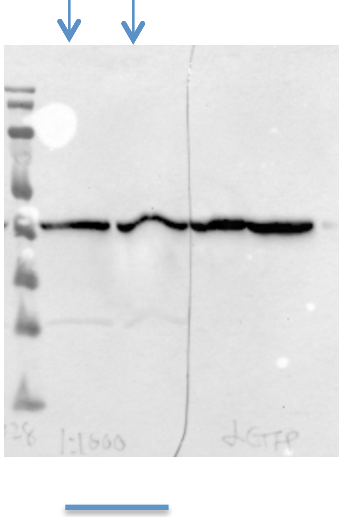

4C, right

Reblot: anti-beta-actin

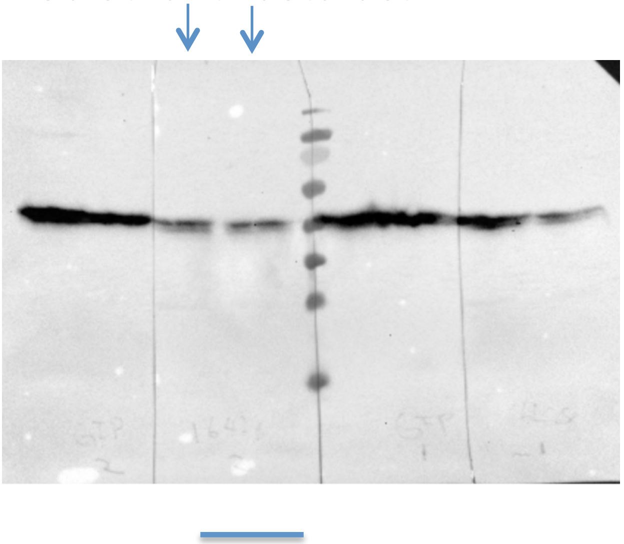

Protein was run on one gel,
membranes were cut with scissors,
and blotted with two different antisera

Figure 4

Figure 4D, top

4D, left

Anti-GFP

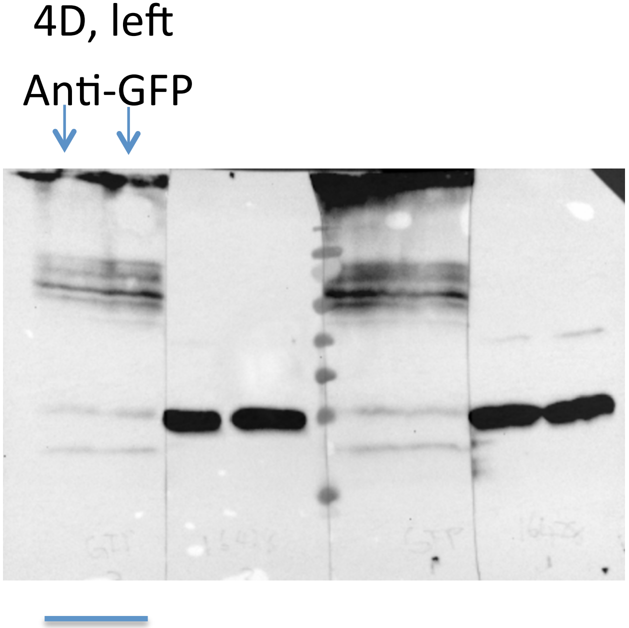

4D, right

Anti-GFP

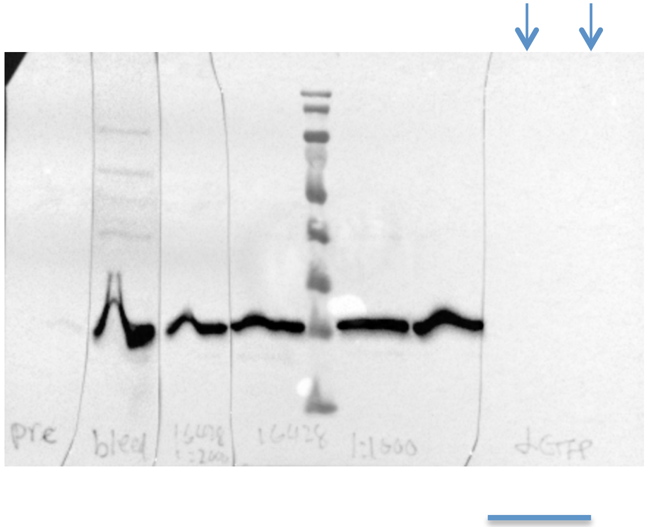

Protein was run on one gel,
membranes were cut with scissors,
and blotted with two different antisera

Figure 4

Figure 4D, bottom

4D, left

Reblot: anti-beta-actin

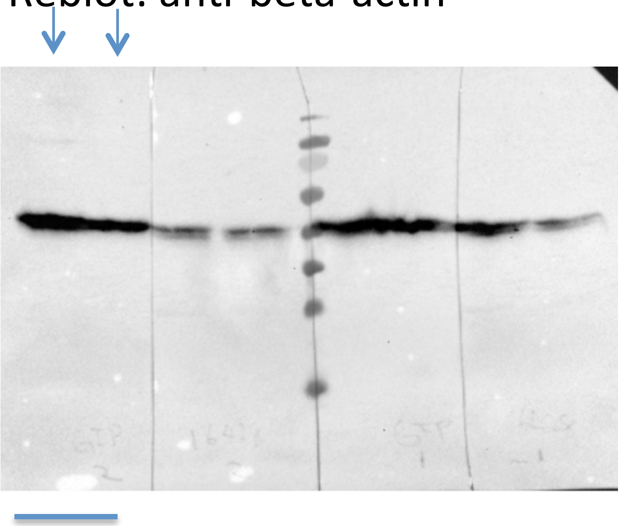

4D, right

Reblot: anti-beta-actin

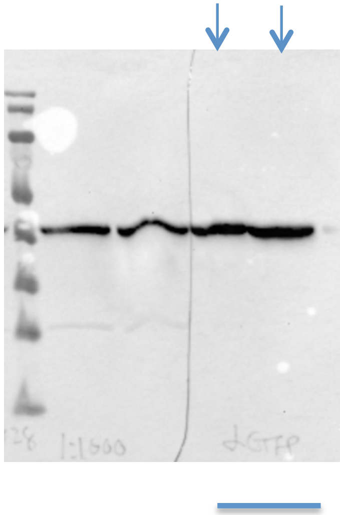

Protein was run on one gel,
membranes were cut with scissors,
and blotted with two different antisera
